# Better target detection in the presence of collinear flankers under high working memory load

**DOI:** 10.3389/fnhum.2014.00821

**Published:** 2014-10-14

**Authors:** Jan W. De Fockert, Jaclyn Leiser

**Affiliations:** Department of Psychology, Goldsmiths, University of LondonLondon, UK

**Keywords:** working memory, selective attention, target detection, Gabor stimuli, collinear flankers

## Abstract

There are multiple ways in which working memory can influence selective attention. Aside from the content-specific effects of working memory on selective attention, whereby attention is more likely to be directed towards information that matches the contents of working memory, the mere level of load on working memory has also been shown to have an effect on selective attention. Specifically, high load on working memory is associated with increased processing of irrelevant information. In most demonstrations of the effect to-date, this has led to impaired target performance, leaving open the possibility that the effect partly reflects an increase in general task difficulty under high load. Here we show that working memory load can result in a performance gain when processing of distracting information aids target performance. The facilitation in the detection of a low-contrast Gabor stimulus in the presence of collinear flanking Gabors was greater when load on a concurrent working memory task was high, compared to low. This finding suggests that working memory can interact with selective attention at an early stage in visual processing.

## Introduction

Over the past decade, evidence has accumulated for a close link between selective attention and working memory (e.g., Awh and Jonides, 2001; Chun, 2011; Gazzaley and Nobre, 2012). An often reported effect is that distractibility in selective attention is enhanced in the context of either high working memory load (e.g., Lavie et al., 2004) or low working memory capacity (e.g., Kane and Engle, 2003; Ahmed and de Fockert, 2012), suggesting that efficient selective attention to task-relevant information relies on the availability of working memory. For example, the interference produced by irrelevant distractor letters flanking a relevant target (Eriksen and Eriksen, 1974) becomes greater under high working memory load (Lavie et al., 2004). Similar modulations of distractor processing as a function of working memory load have been reported in a range of other selective attention tasks (e.g., Lavie and De Fockert, 2005; Pecchinenda and Heil, 2007; Pratt et al., 2011).

At which point in the visual pathway does working memory affect selective processing? The majority of previous work suggesting that high load on a working memory task is associated with greater processing of task-irrelevant information in vision has used response-competition tasks. On those tasks, the effect of working memory load could originate anywhere between initial visual processing of the distractors and final response selection. Certain findings suggest that the effect of working memory on distractor processing occurs relatively early. First, working memory affects processing of distractors that are not associated with a task response (Lavie and De Fockert, 2005; De Fockert and Wu, 2009). Second, load on working memory load increases the attentional blink (Akyürek et al., 2007), an effect that is associated with modulation of an early component in electrophysiology (Akyürek et al., 2010). Finally, neuroimaging work has shown that activity in object-specific areas of the visual cortex, associated with distractor processing, is greater under high working memory load (De Fockert et al., 2001; Kelley and Lavie, 2011). High working memory load can also delay the neural response to a visual target in low-level visual areas in occipital cortex (Scalf et al., 2011).

The first aim of the current study was to examine the role of working memory in early vision processing. We measured the modulation of detection of a low contrast visual stimulus by the presence of high contrast flankers (Polat and Sagi, 1993), an effect that is thought to originate from lateral interactions between neural assemblies in early visual cortex (Gilbert and Wiesel, 1989; Polat et al., 1998; Freeman et al., 2001). When a low contrast Gabor stimulus is presented at fixation, detection thresholds are lower when it is flanked by high contrast, spatially aligned Gabors. In the first study to describe the lateral interactions produced by collinear visual flankers (Polat and Sagi, 1993), a centrally presented target Gabor was flanked by two high contrast flankers at varying target-to-flanker separations. In order for the flankers to facilitate target detection, the flankers have to have the same orientation as the target, be spatially aligned with the target, and occur at a certain target-to-flanker separation (Polat and Sagi, 1993). When those conditions are met, detection thresholds are significantly lower in the presence (vs. absence) of the flankers.

Importantly, the facilitation of target processing by high contrast flankers occurs only when the flankers are attended (Freeman et al., 2001). When observers are presented with a low contrast Gabor target accompanied by four flankers, two of which are aligned with the target and two of which are not, and are made to attend to two of the flankers while ignoring the other two, facilitation effects only occur when the attended flankers are collinear with the target. In other words, although collinear flankers were always present, they only facilitated detection thresholds when they were attended (Freeman et al., 2001). This finding strongly suggests that the high contrast flankers need to receive some attention in order to produce the performance gain on target detection. In the current study, observers were told that the flankers were irrelevant to the task (as their presence was not predictive of the presence of the relevant target), and attention should be focused on a central target stimulus. We argued that observers would be less able to maintain attentional focus on the target, and be more likely to attend to the irrelevant flanking Gabors, when working memory was highly loaded, as the ability to maintain attentional focus on the target location is compromised under high working memory load (e.g., Lavie et al., 2004; De Fockert and Bremner, 2011). As a result, the facilitation effect from collinear flankers should be greater under high working memory load.

In addition to testing whether working memory can modulate the effect of distractors that are assumed to involve early visual processing, our study had a second aim. The prediction that target processing should gain from high working memory load when distractors facilitate target performance forms a key test of the idea that loading working memory increases distraction. The previous evidence has mostly shown performance impairments in selective attention following increases in working memory load, such as slower and less accurate target responses because of greater flanker interference (Lavie et al., 2004), or stronger attentional capture by salient distractor singletons (Lavie and De Fockert, 2005), leaving open the possibility that the effects of high working memory load on performance in attention tasks partly result from an increase in general task difficulty. By contrast, here we anticipated an improvement in target performance under high working memory load, as more attention to the flankers should aid target detection.

## Method

### Participants and stimuli

Thirteen people (mean age, 20 years 5 months) volunteered to participate in the experiment, which was approved by the Department Ethics Committee at Goldsmiths. Sample size was determined on the basis of previous published work with the dual-task paradigm used here, showing that a significant effect of working memory load on distractor processing is obtained with around 12 participants (e.g., Lavie et al., 2004). We tested an additional participant to prevent being underpowered following the exclusion of one participant with poor performance on the working memory task.

The experiment was run in a darkened testing cubicle on a PC running E-Prime (Schneider et al., 2002). Stimuli were displayed on a non-linearized CRT (Mitsubishi Diamond Plus 220) at a viewing distance of approximately 60 cm. Gabor stimuli for the visual detection task were generated using an online E-Prime script^1^. The Gabor stimuli were symmetrical, with a Gaussian envelope and a spatial frequency of seven half-cycles in horizontal orientation. For the target stimuli, stimulus contrast was 0.3% (low contrast), 0.5% (medium contrast), or 0.9% (high contrast). For the flanker stimuli, contrast was either 50% (flankers present) or 0% (flankers absent). Gabor stimuli had a wavelength subtending 0.95° of visual angle, and the diameter of each Gabor stimulus subtended 2.48°. Target stimuli were presented at fixation in the screen center. Flankers were presented left and right of the target at 2.58° target-distractor center-to-center separation (2.7 wavelengths, a target-flanker distance previously shown to produce robust facilitation effects; Polat and Sagi, 1993), so that the entire stimulus array subtended 7.63° horizontally.

**Figure 1:**
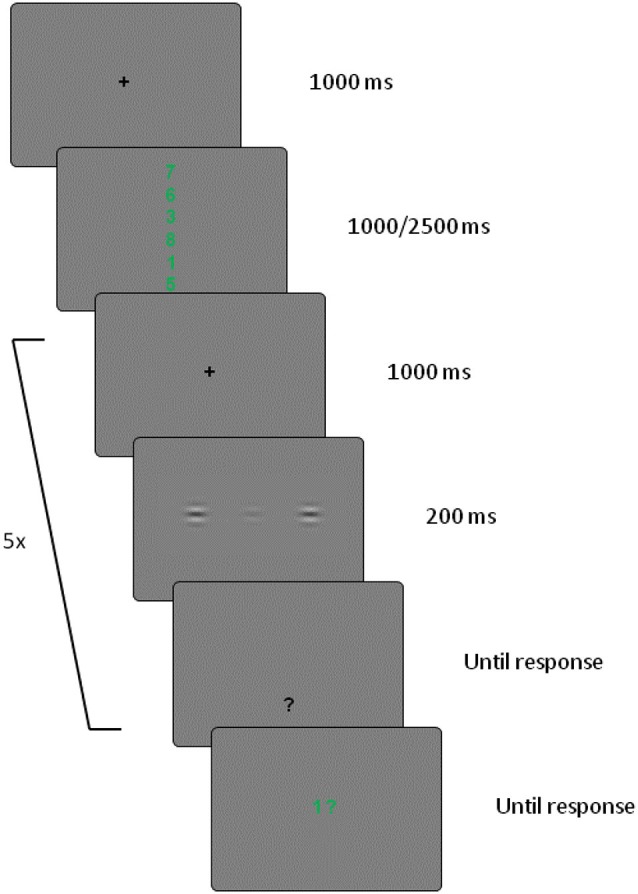
**Example of a high working memory load trial**. Low working memory load trials were the same, apart from the memory set which contained only one digit presented for 1000 ms. There were five target detection displays for each working memory trial (images not to scale, target contrast enhanced for display purposes).

For the working memory task, a memory set consisting of either one (low load) or six (high load) digits was presented in green at the start of each trial. The single digit in the low load condition was presented at fixation. The six digits in the high load conditions were presented in a vertical line centered at fixation (to prevent cueing attention to the Gabor flanker locations which may occur following a horizontally presented memory set). Each digit subtended 0.38° horizontally and 0.57° vertically, and the entire high load set subtended 5.25° vertically. The memory probe was a single green digit with a question mark, presented at fixation. All stimuli were presented on a gray background (RGB values, 128,128,128), see Figure 1 for example stimulus displays.

### Procedure

Each trial began with a 1000 ms central fixation screen (a black plus sign), followed by the memory set. The memory set consisted of one digit presented for 1000 ms in the low working memory load condition, and six digits presented for 2500 ms in the high load condition. The presentation durations of the low and high memory load sets allowed participants to rehearse the set at least once before presentation of the target detection trials. Participants had to memorize the set until the end of the trial. Next, the visual detection displays were presented. Each working memory trial contained five detection trials (to increase the amount of detection data collected for each working memory trial). Detection displays were presented for 200 ms, followed by a response screen consisting of a black question mark presented 5.44° below fixation (to prevent it from masking the preceding target stimulus), that remained visible until a response had been recorded. Participants were asked to press one of two keys on the numerical keypad with their right hand to indicate whether they thought the visual target stimulus had been present (press <2>) or absent (press <0>). On half the trials, the target was present, with equal proportions of each target contrast condition. On the other half of the trials, the target was absent. Different detection trial conditions were presented within each working memory trial. Accuracy feedback was given in the form of a tone following an incorrect response. After the final detection trial, a single memory probe digit was presented, and the participant had to use the same two keys as for the detection task to indicate whether or not the probe had been present in the set for that trial. On half of the working memory trials, a “present” response was correct. A feedback tone was presented following an incorrect response to the memory probe. Participants first received 32 practice trials on the detection task alone, on which the Gabor stimuli were presented until response. Next, they received five combined working memory/detection trials with high load working memory, with the same presentation durations as in the experimental trials. Two experimental blocks were then presented, one with low and one with high working memory load (order counterbalanced between participants), each containing 32 working memory trials (160 detection trials per working memory load condition).

## Results

Data from one participant, whose accuracy on the working memory task was below chance under high working memory load, were excluded. For the remaining 12 participants, responses to the working memory probe were analyzed first, which confirmed that the manipulation was effective in loading working memory. Mean response times were faster in the low working memory load condition (*M* = 1260) compared to the high working memory load condition (*M* = 1562; *t*_(11)_ = 2.61, SEM = 115.8, *p* < 0.025, *d* = 0.78). Mean accuracy rates were also higher in the low working memory load condition (*M* = 0.841) compared to the high working memory load condition (*M* = 0.815), although this difference was not significant (*t* < 1).

**Figure 2:**
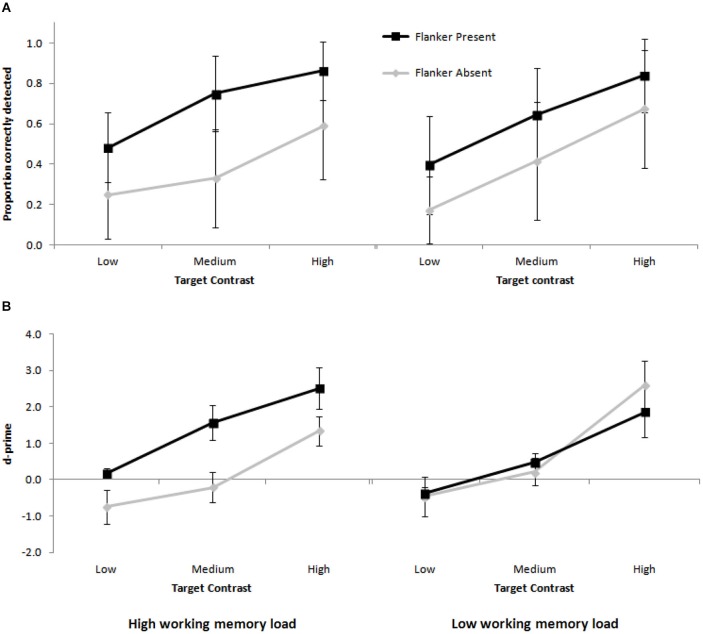
**Mean proportion correctly detected targets (A) and d-prime scores (B), as a function of target contrast, flanker presence, and working memory load**. Error bars represent between-subject standard error of the mean.

**Figure 3:**
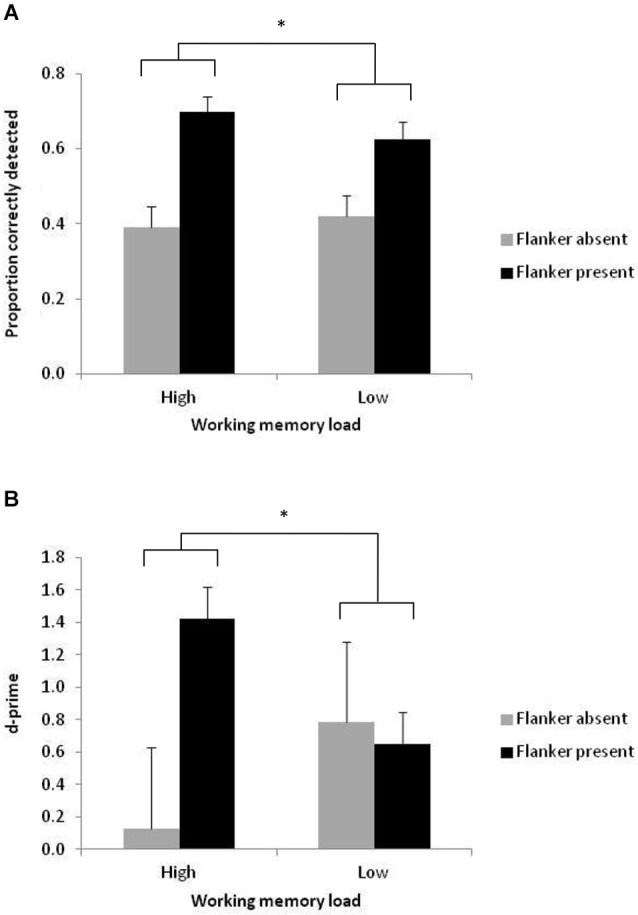
**The interaction between flanker presence and working memory load in mean proportion correctly detected targets (A) and d-prime scores (B)**. Error bars represent between-subject standard error of the mean. * *p* < 0.05.

Next, the probability to correctly detect a present Gabor patch was analyzed in a three (target contrast: low, medium, high) by two (flanker condition: present, absent) by two (working memory load: low, high) fully within-subjects Analysis of Variance (ANOVA; see Figure 2A). Only trials on which the working memory response was correct were included in this analysis. Stimulus visibility was successfully manipulated, as shown by a main effect of target contrast, *F*_(1,11)_ = 42.03, MSe = 0.049, *p* < 0.001, $\eta_{p}^{2}$ = 0.793. Targets were least likely to be deemed present when they had low contrast (*M* = 0.326) compared to medium contrast (*M* = 0.535; *t*_(11)_ = 5.86, SEM = 0.036, *p* < 0.001, *d* = 1.74) and high contrast (*M* = 0.741; *t*_(11)_ = 7.18, SEM = 0.058, *p* < 0.001, *d* = 2.09), and less likely to be deemed present when they had medium contrast compared to high contrast (*t*_(11)_ = 5.26, SEM = 0.039, *p* < 0.001, *d* = 1.52). The flanker facilitation effect (Polat and Sagi, 1993) was replicated, as shown by a significant main effect of flanker condition, *F*_(1,11)_ = 18.92, MSe = 0.126, *p* < 0.01, $\eta_{p}^{2}$ = 0.632. Targets were more likely to be deemed present when the flankers were present (*M* = 0.662) compared to absent (*M* = 0.405). There was no main effect of working memory load (*F* < 1), but crucially, there was a significant two-way interaction between working memory load and flanker condition, *F*_(1,11)_ = 4.93, MSe = 0.020, *p* < 0.05, $\eta_{p}^{2}$ = 0.310 (see Figure 3A). Planned follow-up tests (Bonferroni corrected) showed that the presence of the flankers led to a greater improvement in detection rates under high working memory load (from 0.389 to 0.699 in flanker absent and present conditions, respectively, *t*_(11)_ = 4.47, SEM = 0.069, *p* < 0.001, *d* = 1.32) than under low working memory load (from 0.421 to 0.626, *t*_(11)_ = 3.57, SEM = 0.057, *p* < 0.01, *d* = 1.04). No other effects were significant.

To confirm that the pattern of results in the main analysis did not reflect changes in response bias, d-prime scores were also computed as a function of target contrast, flanker condition and working memory load (see Figure 2B). Data were excluded from the d-prime analysis for one participant, whose overall d-prime score constituted an outlier (mean d-prime 1.82 standard deviations, with one of the conditions over four standard deviations away from the group mean). The d-prime scores showed the same pattern as the detection rates, with a main effect of target contrast, *F*_(1,10)_ = 19.16, MSe = 3.50, *p* < 0.001, $\eta_{p}^{2}$ = 0.657. D-prime scores were greater for high contrast targets (*M* = 2.08), compared to medium (*M* = 0.51, *t*_(10)_ = 3.6, SEM = 0.437, *p* < 0.01, *d* = 1.14) and low contrast targets (*M* = −0.36, *t*_(10)_ = 5.15, SEM = 0.473, *p* < 0.001, *d* = 1.69). D-prime was greater in medium, compared with low contrast targets, *t*_(10)_ = 3.44, SEM = 0.252, *p* < 0.01, *d* = 1.14. D-prime scores were also greater when the flankers were present (*M* = 1.03) than when they were absent (*M* = 0.45), although the main effect of flanker condition was not significant (*F*_(1,11)_ = 2.61, MSe = 4.25, *p* = 0.138, $\eta_{p}^{2}$ = 0.207). The main effect of working memory load was not significant (*F* < 1), but importantly, there was again a significant interaction between working memory load and flanker condition, *F*_(1,10)_ = 5.44, MSe = 3.09, *p* < 0.05, $\eta_{p}^{2}$ = 0.353 (see Figure 3B). Planned follow-up tests (Bonferroni corrected) showed that under high working memory load, there was a significant difference in d-prime between flanker present (*M* = 1.42) and absent displays (*M* = 0.13, *t*_(10)_ = 3.25, SEM = 0.398, *p* < 0.01, *d* = 0.98). Under low working memory load, d-prime scores were not significantly affected by the presence of the flankers (flankers present, *M* = 0.65; flankers absent, *M* = 0.78; *t* < 1). No other effects were significant.

## Discussion

Target detection accuracy was significantly better in the presence of peripheral collinear flankers, and more so when concurrent load on working memory was high. The enhanced effect of the irrelevant flankers on target detection performance when the working memory task was relatively difficult is in line with two pieces of previous evidence. First, only collinear flankers that receive attentional processing facilitate target visibility (Freeman et al., 2001). Second, the extent of attentional processing of non-target information depends on the availability of working memory during selective attention (Lavie et al., 2004). Together, these prior findings suggest that making working memory unavailable for selection by increasing the load on a working memory task should increase the processing of the collinear flankers, leading to better target detection. This is what we found.

Performance improvements under high working memory load should be obtainable in other flanker tasks, whenever the distracting information is compatible with the current target. Indeed, in a previous study that measured both facilitation and interference effects from irrelevant distractor letters under varying levels of working memory load (Lavie et al., 2004, Experiment 1), both facilitation and interference were increased under high working memory load. However, flanker compatibility effects are more commonly computed by contrasting performance on trials with compatible and incompatible flankers, making it impossible to distinguish facilitation from interference. Moreover, in many response-competition tasks, such as the flanker task and the Stroop task, facilitation and interference are not symmetrical, and interference effects are generally larger than facilitation effects (e.g., Lindsay and Jacoby, 1994). In those paradigms, interference effects may therefore be more likely than facilitation effects to be modulated by working memory load.

There are a few previous demonstrations that performance can benefit in conditions when working memory is relatively unavailable for selective attention. When performing a demanding visual task, such as comparing the sizes of two lines with very similar lengths, an unexpected visual stimulus presented close to fixation often remains undetected (Rock et al., 1992). Such inattentional blindness is reduced (i.e., detection of the unexpected item is better) under high working memory load, presumably because selective attention is less efficiently focused on the relevant lines (De Fockert and Bremner, 2011). Inattentional blindness, however, concerns information other than the hitherto task-relevant lines, and therefore the release from inattentional blindness by working memory load does not involve a change in target performance, like we found in the current study. Other work has found that loading working memory can aid target performance, not because the processing of task-compatible distractors becomes more likely under high load, like we found here, but because high load can lead to a reduction in distractor processing, as long as the content of the working memory task has greater overlap with the distractor than with the target (Kim et al., 2005; Park et al., 2007). The current findings are therefore the first demonstration that loading working memory can facilitate perception following greater attention to irrelevant information.

Previous work has shown that attention can enhance detection of the type of stimulus that was used here (e.g., Cameron et al., 2002; Pestilli and Carrasco, 2005). In the absence of the flankers, we might therefore have expected target detection to be better under low (vs. high) working memory load, assuming that attention would be better focused on the target under low working memory load. Although in the flanker absent conditions, performance was indeed somewhat better under low, compared to high working memory load both in terms of accurate detection (low load, *M* = 0.42; high load, *M* = 0.39) and d-prime (low load, *M* = 0.78; high load, *M* = 0.13), neither of these differences reached statistical significance. In other words, whereas our manipulation of working memory load had the predicted effect of modulating target detection in the presence of the flankers, high working memory load did not reliably impair target detection when the flankers were absent.

The finding that in the flanker absent conditions, target detection was similar under low and high working memory load is also important in order to eliminate an alternative (yet invalid) explanation of the better target detection in the presence of flankers under high load. Following standard practice in the literature using the combined working memory/selective attention paradigm, working memory load was manipulated in separate blocks. It could therefore be argued that participants were simply more attentive in the high load condition, which in turn led to their better target detection when flankers were present. If this were the case, however, target detection should also have been better under high (vs. low) load in the absence of the flankers, which was clearly not the case: if anything, target detection without flankers was somewhat better under low working memory load, which is in line with recent findings showing that the subjective visibility of a briefly presented number stimulus is reduced as load on a concurrent working memory task increases (De Loof et al., 2013).

The lateral interactions that produce the flanker facilitation effects shown here are thought to occur in primary visual cortex (Gilbert and Wiesel, 1989; Polat et al., 1998), so these findings suggest that working memory may affect the processing of task-irrelevant information at an early stage. Unlike previous demonstrations showing greater distractor processing in a context of high (vs. low) working memory load, where the effect may have been due to greater response competition (e.g., Lavie et al., 2004), the improved target detection observed under high working memory load in the current study is likely to reflect greater perception of the peripheral flankers. Together with other previous findings suggesting that working memory affects the perception of to-be-ignored distractors (Lavie and De Fockert, 2005; De Fockert and Wu, 2009; Kelley and Lavie, 2011), our results show that working memory can affect the prioritization of information at an early stage in visual processing. The finding that load on a working memory task that involved maintaining sets of digits interacted with processing of visual distractors in a simple target detection task suggests that the link between working memory and low-level visual processing is indirect in this case, in that it does not involve content-specific interactions between working memory and perception. Instead, working memory and perceptual processing are more likely to interact in terms of resource demands in this case, such that general working memory resources are required to sustain focused visual selection.

## Conflict of interest statement

The authors declare that the research was conducted in the absence of any commercial or financial relationships that could be construed as a potential conflict of interest.

## Footnotes

^1^http://vision.psy.unipd.it/equipment.htm
